# Influence of Temperature on Motor Behaviors in Newborn Opossums (*Monodelphis domestica*): An *In Vitro* Study

**DOI:** 10.1523/ENEURO.0347-18.2019

**Published:** 2019-06-04

**Authors:** Edith Corriveau-Parenteau, Ariane Beauvais, Annie Angers, Jean-François Pflieger

**Affiliations:** Université de Montréal, Montréal, Quebec H3C 3J7, Canada

**Keywords:** development, motor behaviors, thermosensation, trigeminal system, TRPM8

## Abstract

External thermosensation is crucial to regulate animal behavior and homeostasis, but the development of the mammalian thermosensory system is not well known. We investigated whether temperature could play a role in the control of movements in a mammalian model born very immature, the opossum (*Monodelphis domestica*). Like other marsupials, at birth the opossum performs alternate and rhythmic movements with its forelimbs (FLs) to reach a teat where it attaches in order to continue its development. It was shown that FL movements can be induced by mechanical stimulation of the snout in *in vitro* preparations of newborns consisting of the neuraxis with skin and FLs intact. In the present study, we used puff ejections of cold, neutral (bath temperature) and hot liquid directed toward the snout to induce FL responses in such preparations. Either the responses were visually observed under a microscope or triceps muscle activity was recorded. Cold liquid systematically induced FL movements and triceps contractions, but neutral and hot temperatures were less potent to do so. Sections of the trigeminal nerves and removal of the facial skin diminished responses to cold and nearly abolished those to hot and neutral stimulations. Transient receptor potential melastatin 8 (TRPM8) being the major cold receptor cation channel in adult mammals, we employed immunohistochemistry and reverse transcription-polymerase chain reaction (RT-PCR) to test for its expression, but found that it is not expressed before 13 postnatal days. Overall our results indicate that cold thermosensation exerts a strong influence on motor behaviors in newborn opossums.

## Visual Abstract

**Figure:**
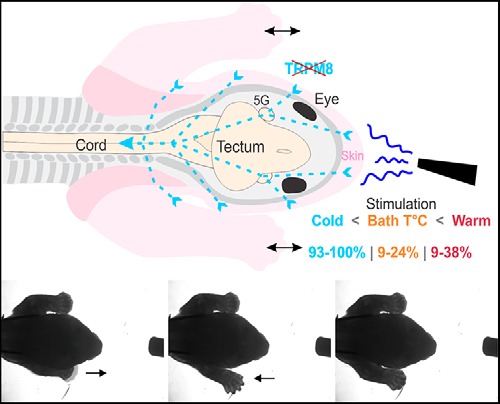

## Significance Statement

External thermosensation is crucial for survival, but its development in mammals is not well understood, particularly at the systemic level. Herein, we tested whether temperature perceived by the face influences motor behaviors in newborn opossums, a marsupial with a gestation period one week shorter than rodents of comparable size, thus offering access to early developmental stages. We found that cold temperatures systematically induced forelimb (FL) motor responses, but neutral and hot temperatures rarely did so. Moreover, in newborn opossums, cold thermosensation does not involve the major cold receptor in adult mammals, transient receptor potential melastatin 8 (TRPM8). Cold avoidance may be important to sustain motor behaviors of newborn marsupials, when they must find a teat and attach to it to pursue their development.

## Introduction

Changes in external temperature activate thermosensory receptors on peripheral nerve endings of sensory neurons located in spinal dorsal root ganglia (DRG) and cephalic ganglia. Studies focused on the identification and physiologic properties of these receptors revealed that they belong mainly to cationic channels of the transient receptor potential (TRP) family (for review, see Schepers and Ringkamp, 2010; Vriens et al., 2014). ThermoTRPs are also activated by chemical compounds. Those which have been best characterized so far are the heat and capsaicin receptor TRPV1, and the cold and menthol receptor TRP melastatin 8 (TRPM8; Caterina et al., 1997; McKemy et al., 2002; Peier et al., 2002a). Other known mammalian thermoTRPs include TRPV3-4, TRPM3, and TRPA1 (Güler et al., 2002; Peier et al., 2002b; Watanabe et al., 2002; Story et al., 2003; Vriens et al., 2011), but only TRPM8 was shown unambiguously to a have major role in temperature sensing *in vivo* (Bautista et al., 2007; Dhaka et al., 2007; Knowlton et al., 2013). The molecular properties of these channels have been well documented, but few studies address how the central nervous system processes temperature information (Pogorzala et al., 2013; Ran et al., 2016; Yarmolinsky et al., 2016).

Thermosensation in immature mammals was mostly studied on the spinal cord and DRG. During mouse embryonic development, the expression of TRPV1 in DRG cells starts around 12.5 d of gestation (E12.5), followed by the expression of TRPM8 around E16.5 (Tamura et al., 2005; Hjerling-Leffler et al., 2007). Bath application of capsaicin or menthol on *in vitro* isolated spinal cord of wild-type and transgenic neonatal mice showed that sensory afferents expressing TRPV1 or TRPM8, respectively, modulate the activity of spinal networks generating locomotor rhythms (Mandadi et al., 2009, 2013); in similar *in vitro* preparations of neonatal rats, but with one hindlimb left attached, ongoing locomotor-like rhythm could be affected by application of capsaicin, heated- or cooled-liquid on the hindpaw (Mandadi and Whelan, 2009). Infrared radiant-heat applied to sacro-caudal dermatomes can induce locomotor-like activity in *in vitro* semi-intact preparations of neonatal rats (Blivis et al., 2007).

Embryos of placental mammals, like rodents or humans, develop in the temperature-stable environment of the womb and are exposed to temperature variations relatively late in their development. By contrast, marsupial mammals, like kangaroos and opossums, are born prematurely, and it has been postulated that thermosensation may already be functional at birth and affect their behaviors (Langworthy, 1928; Nelson and Gemmell, 2004).

To test this hypothesis, we investigated whether facial thermosensation is functional at early stages of maturation in gray short-tailed opossums, *Monodelphis domestica*. The newborn opossum is very immature, approximately equivalent to E11.5–E13.5 mouse or rat embryos (Cabana, 2000; Smith, 2001), but performs alternate and rhythmic movements with its forelimbs (FLs) to climb on the mother’s belly and reach a teat where it attaches to pursue its development. Cephalic sensory inputs must be involved to trigger these movements and induce the attachment to the teat. We focused our study on the face as it has been demonstrated that the trigeminal afferents, which relay facial mechanosensory, nociceptive and thermosensory inputs in adult mammals (Capra and Dessem, 1992; Viana, 2011), are functional in newborn opossums and act strongly on limb motricity (Adadja et al., 2013; Desmarais et al., 2016).

The small size and immaturity of newborn opossums allow the making of semi-intact *in vitro* preparations with brainstem and spinal cord left in the carcass and with the limbs and tail attached (Lavallée and Pflieger, 2009). In such preparations, we stimulated the skin of the head with puff ejections of cooled, warmed or bath temperature solutions. Motor responses were recorded as movements of one or both FL or as contractions of the triceps muscles. Cold stimulations steadily induced motor responses, while bath and hot temperatures did so far less regularly. Complete transections of the trigeminal nerve (5N) diminished the intensity of motor responses to cold and hot stimuli, supporting a role for the trigeminal system in mediating thermosensation. Reverse transcription-polymerase chain reaction (RT-PCR) and immunohistochemistry experiments showed that TRPM8 is not expressed before postnatal day (P)13. This study thus demonstrates that newborn opossums are more responsive to cold than to warm temperature, which may induce an avoidance behavior to cold. Preliminary results have been published in abstract form (Corriveau-Parenteau et al., 2016, 2017).

## Materials and Methods

### Animal care

A colony of gray short tailed opossums (*M. domestica*) is maintained at the institution’s animal facility according to the guidelines developed by Fadem et al. (1982; for further details on animal care and breeding, see VandeBerg and Williams-Blangero, 2010; Desmarais et al., 2016). The present protocol follows the guidelines of the Canadian Council on Animal Care and was approved by the University of Montréal animal ethics committee.

### *In vitro* preparations

**Figure 1.**
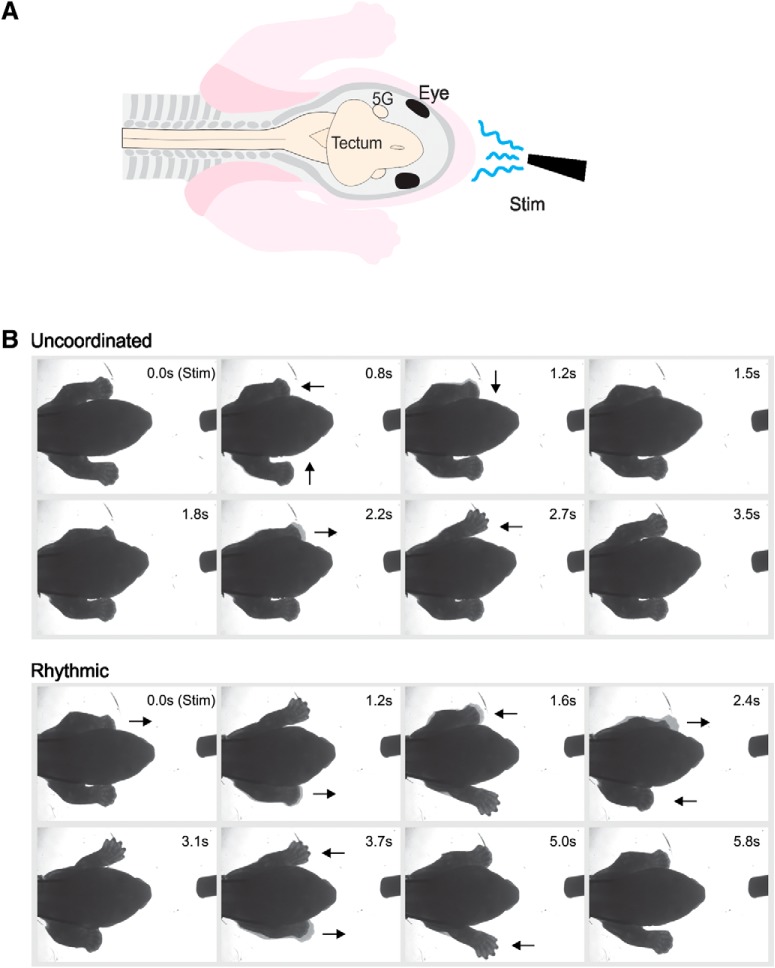
FL behavioral observation experiments. ***A***, Schematic representation of the *in vitro* preparation. The specimen has skin over all its face, neck and FL, and the FL are free to move. 5G, trigeminal ganglion; Stim, stimulation. ***B***, Serial photographs taken from video of either uncoordinated (upper rows) or rhythmic (left-right alternation; lower rows) responses after stimulation. Arrows indicate the direction of paw movements.

**Figure 2.**
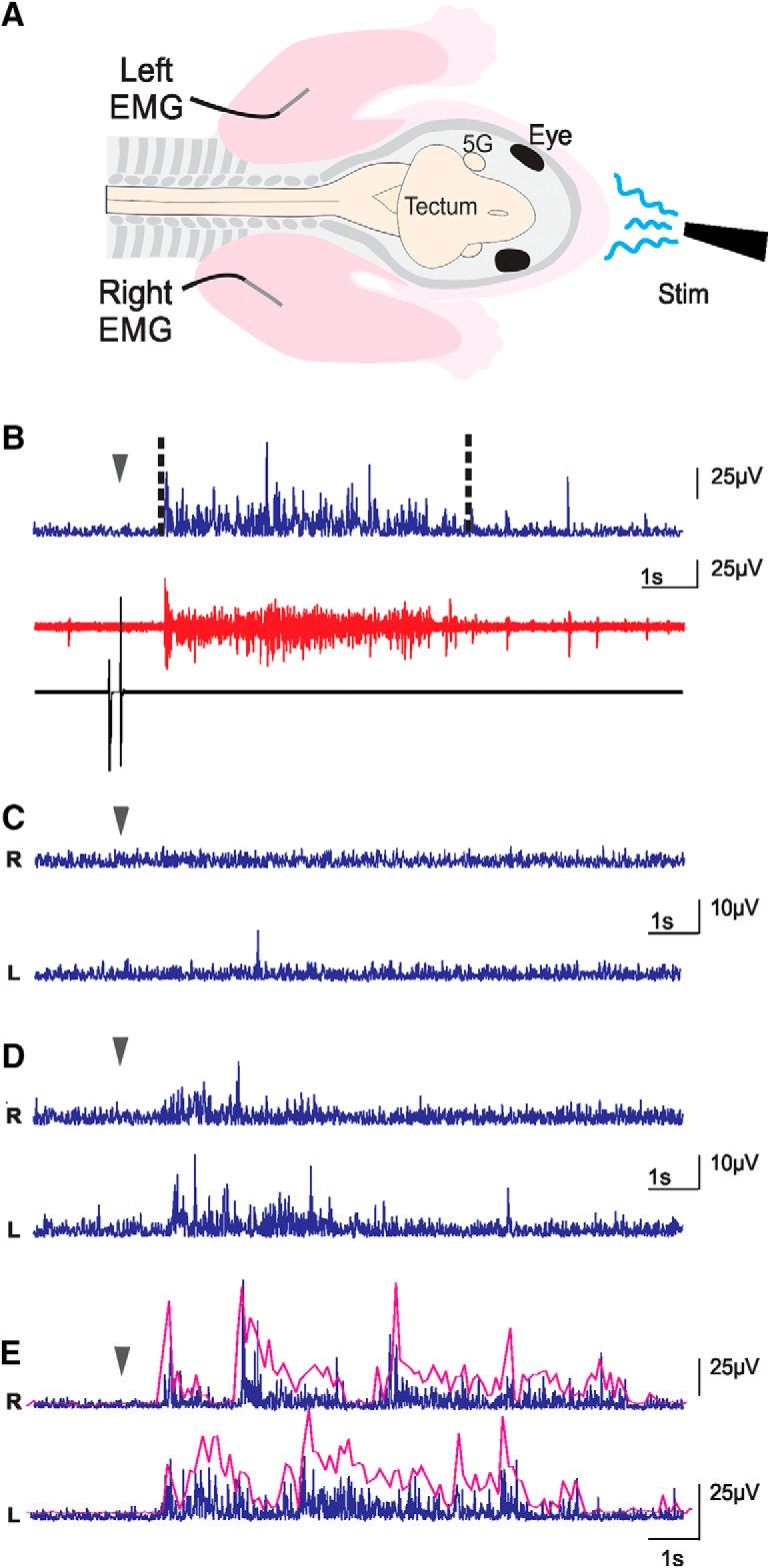
EMG experiments. ***A***, Schematic representation of the preparations used in EMG recordings. FL were pinned on the bath floor (bath not illustrated) so as to limit movements. Skin was removed on the neck and FL, and EMG electrodes were implanted in triceps muscles. 5G, trigeminal ganglion; Stim, stimulation. ***B***, Muscle activity following a stimulation. Bottom black trace, stimulation artifact produced by the pedal; red trace, raw recording from one EMG; blue trace, same trace as in red, but rectified and with a reduced sampling rate. The dashed lines delimitate the duration of the response used for analysis. ***C***–***E***, Processed traces exemplifying reactions to stimulation of the left (L) and right (R) triceps muscles of the same animal: no-response (***C***), uncoordinated response (***D***), and rhythmic response (***E***). In ***B–E***, the arrowheads indicate the beginning of the stimulation. The magenta lines in ***E*** are envelopes of burst responses highlighting the rhythmical alternation (not to scale with EMG traces).

All physiological experiments presented here were conducted on *in vitro* preparations of both male and female opossums (*n* = 51) aged from P0 (day of birth) to P4. In most cases, the specimens were deeply anesthetized by hypothermia but four were anesthetized by isoflurane inhalation. All animals were eviscerated with microscissors before being pinned dorsal side up on a Sylgard-lined Petri dish filled with a physiologic solution (125 mM NaCl, 3 mM KCl, 25 mM NaHCO_3_, 1 mM NaH_2_PO_4_, 1 mM MgCl_2_, 2mM CaCl_2_, and 15 mM glucose; equilibrated with 95%O_2_–5%CO_2_, pH7.4; adapted from Nicholls et al., 1990; Stewart et al., 1991). Under a surgical microscope (Olympus sz61), the skin was removed from over the frontal cartilage down to mid-thoracic level, leaving as much skin as possible on the snout and chin, as well as on the neck and FL so as not to alter FL movements for the behavioral experiments (Fig. 1*A*). The skin over the neck and arm was removed for the EMG recordings (Fig. 2*A*). The hindquarters and tail were kept intact. A craniotomy and a laminectomy were performed to expose the neuraxis and a decerebration was done by dissecting out the diencephalon and telencephalon. The preparations were then pinned for recording and left undisturbed for 1.5–2 h before beginning of experiments. During dissection and experimentation, a peristaltic pump (323S, Watson-Marlow) was used to continually superfuse the physiologic solution at room temperature (25 ± 1°C or 22 ± 1°C) in the dishes. The solution was kept at ambient temperature because newborn marsupials are ectothermic. Indeed, their thermoregulatory capacity develops in parallel with fur (Russell, 1982) that begins to appear around the end of the second postnatal week in opossums (Pflieger et al., 1996; VandeBerg and Williams-Blangero, 2010). Also motor activity of *in vitro* preparations of newborn opossums has been reported as stronger when the bath is at ambient temperature than when it is warmed (Nicholls et al., 1990).

### Stimulations

A stimulation consisted of the manual ejection with a syringe (1 ml; 18-G needle) of a small volume (0.1–0.3 ml) of physiologic solution directed toward the face. Each specimen was stimulated 10 times at a given temperature, with 40 to 120 s interval between stimulations, before testing a different temperature. The number of stimuli was chosen to facilitate analysis and the interval to allow adequate time to refill the syringe. The temperatures tested are indicated in the Results section as an average; they may have varied by ±1°C. The solution at neutral temperature was taken directly from the bath without disturbing the specimen, while cold and hot solutions were kept separately in beakers placed either on ice or over a heating plate. As nothing was known about thermosensory capacity and expression of thermoreceptors in the opossum before our experiments, we did not use temperature ranges corresponding to specific receptors but temperatures closer or further from the neutral one. Preliminary experiments (data not shown) suggested that the sequence order of temperature used for stimulation did not affect the response rates. Therefore, in the experiments reported here, the specimens were generally stimulated with temperatures from the coldest to the warmest.

### Observations of FL movements

The specimens (*n* = 38) were pinned to the bath substrate with the FL free to move. FL movements before and following stimulations were observed visually under the surgical microscope and described either as no response (Movie 1) or response, which consisted of movements of the FL. The latter were further classified as uncoordinated (single extension of one or both FL without obvious coordination or rhythm; Fig. 1*B*, upper rows; Movie 2) or rhythmic (locomotor-like, alternate extensions of the left and right FL; Fig. 1*B*, lower rows; Movie 3). In some experiments, a digital camera (Micropublisher 3.3 RTV; Qimaging) was mounted on the surgical microscope and connected to a computer to allow video recording (12.8 frames/s) with Image-Pro Plus 7.0 (Media Cybernetics).

Movie 1. Ejection of liquid at bath temperature (22°C) toward the snout of an *in vitro* preparation of a P1 opossum do not induce motor response. The stimulation starts at the beginning of the video. 10.1523/ENEURO.0347-18.2019.video.1

Movie 2. Uncoordinated response of the limbs induced by ejection of cold liquid (4°C) toward the snout of an *in vitro* preparation of a P1 opossum. The stimulation starts at the beginning of the video. 10.1523/ENEURO.0347-18.2019.video.2

Movie 3. Rhythmic response of the limbs induced by ejection of cold liquid (4°C) toward the snout of an *in vitro* preparation of a P1 opossum. The stimulation starts at the beginning of the video. 10.1523/ENEURO.0347-18.2019.video.3

### Electromyographic (EMG) recordings of triceps brachii

Newborn opossums measure ∼10 mm from crown to rump, with the head ∼4 mm long and 3.5 mm wide, and their arms 1.0–1.5 mm in diameter. The small size of the arms prevented us to record EMG on freely moving FL. Therefore, additional *in vitro* preparations (*n* = 13) with the FL securely pinned to the substrate, to minimize body movements, were used to record contractions of the triceps brachii muscles, which are FL extensors. The skin from the neck to the elbow of both FL was removed and a Teflon-coated silver wire (wire diameter: 76.2 μm; total diameter: 139.7 μm; A-M Systems Inc) was inserted in the triceps muscle of each FL (Fig. 2*A*). Because of the triceps small size at the ages studied it is possible that activity of nearby muscles was also recorded. The electrodes were maintained in place by a small piece of Teflon tape pressed over the arm. As both electrodes did not remain inserted in all specimens, results were considered independently for each FL. Each electrode was connected to a high impedance module (HZP, Grass). An artifact marking the start of the stimulation was generated with a pedal by the experimenter (Linemaster Switch Corporation). The signals from the electrodes and the pedal were amplified (10×; CP511, Grass Technologies amplifiers) and filtered (bandwidth: 30 Hz to 3 kHz; 60-Hz line filter) before being digitized (Digidata 1322A, Molecular Devices) and recorded at a sampling rate of 11.1 kHz with Clampex 9.2 (Molecular Devices).

For analysis, the EMG traces were rectified, and the sampling rate reduced to 100 Hz using Clampfit 10.6 (Molecular Devices). The average baseline trace, as measured over 7–10 s before stimulation, was adjusted at 0 V. The latency and amplitude of the responses were measured on the resulting trace with the same software. A response consisted of a positive trace deviation over 4.5× the baseline until it declined under this threshold for at least 3 s (Fig. 2*B*). Latency was measured from the peak of the artifact to the onset of the EMG response, and its amplitude consisted of the area under the trace for the response duration (Fig. 2*B*).

### Immunohistochemistry and RT-PCR

Since the behavioral observations and EMG recordings showed a strong effect of cold but not of hot temperature stimulations the following experiments focused on the cold receptor TRPM8. These experiments were performed on freshly prepared specimens and not *in vitro* preparations because the time spent in the bath may have altered the quality of the tissues.

Specimens aged P0/P1 (*n* = 4), P5 (*n* = 3), P9 (*n* = 3), and P13/14 (*n* = 6) were deeply anesthetized by hypothermia and decapitated. The heads were immersed in 4% paraformaldehyde for 48 h followed by 30% sucrose for 24–48 h. They were then embedded in optimal cutting compound Tissue Tek (Sakura) and sectioned transversally at 20 µm with a cryostat (Leica CM3050S). The sections were collected on Superfrost slides (Fisher) and allowed to dry overnight before being washed with a 0.05 M Tris buffered solution (TBST; 15% saline, 3% Triton X-100, pH 7.4) containing 5% normal goat serum for 1 h at room temperature. They were then incubated with primary anti-TRPM8 polyclonal antibodies produced in rabbit (1:100 in TBST, Santa Cruz Biotechnologies D-25) for 24 h at 4°C. The sections were rinsed with TBST and incubated with a goat anti-rabbit IgG H&L secondary antibody coupled with Alexa fluor 488 (1:400 in TBST; Santa Cruz Biotechnologies 516606 or Abcam ab150077) for 2–3 h at room temperature. The sections were rinsed thrice with TBST before being mounted with a coverslip using Fluoromount G (Southern Biotech). They were observed with a fluorescence microscope (Nikon ECLIPSE 50i) using a FITC filter. Photographs were acquired with a digital camera (Nikon DS-2Mv) and saved on a computer using NIS-Elements F3.0 (Nikon) imaging software. When needed, adjustment of contrast, luminosity and color was done using Corel PhotoPaint X8.

To verify whether the polyclonal antibodies used for immunohistochemistry raised against a peptide mapping near the C-terminus of human TRPM8 were also specific for the opossum TRPM8, we used RT-PCR on additional specimens aged P0/1 (*n* = 3), P8 (*n* = 1), and P11/12 (*n* = 3). They were deeply anesthetized by hypothermia, decapitated, and the heads were collected. Since spermatozoa express TRPM8 in vertebrates (De Blas et al., 2009; Martínez-López et al., 2011; Majhi et al., 2015), one adult male opossum was deeply anesthetized by isoflurane until it became unresponsive to pinching of the paws and ears. It was then decapitated and its testes were collected to be used as positive control. The heads and testes were immersed in extraction buffer (RLT; QIAGEN) and homogenized with a rotor-stator. Tissues were then treated with proteinase K and DNase I before RNA isolation with RNeasy mini kit (QIAGEN). Total RNA was used for reverse transcription to cDNA using Superscript IV (Invitrogen) and oligo-dT_20_ according to the manufacturer’s instructions. The resulting cDNA was then amplified by PCR with specific primers for TRPM8 and glyceraldehyde-3-phosphate dehydrogenase (GAPDH; Table 1). PCR consisted of 5-min preheating (94°C), followed by 37 cycles of amplification [94°C for 30 s, 56°C (GAPDH) or 58°C (TRPM8) for 30 s, and 72°C for 30 s] and ended with a final extension at 72°C for 10 min. Migration of the PCR product was done on a 1% agarose gel for 30 min at 120 V. A photo was taken using a digital camera (Fusion FX, Vilber Lourmat, MBI Lab Equipment) and examined with FusionCapt Advance Solo 4 16.08a software.

**Table 1.** *M. domestica* specific primers used in RT-PCR experiments

Gene	Sequence (5’-3’)
GAPDH	Forward: TAAATGGGGAGATGCTGGAG
	Reverse: GCCAGCATCGAAGGTAGAAG
TRPM8	Forward: GGTCATTTGGGAGCAGACGA
	Reverse: ATCCATGAGCAGCACGTAGG

### Statistical analysis

Firstly, the percentages of FL movements obtained following stimulations at a given temperature in each specimen were averaged and, secondly, the results from all specimens were pooled. As for the EMG, amplitudes for a given muscle at a given temperature were first expressed as a percentage of the maximal response obtained for the whole sets of stimulations. These percentages were then averaged for this muscle before the data from all muscles were pooled. The results are given as mean ± SEM.

A D’Agostino and Pearson normality test was performed systematically before statistical analysis to determine whether the above values followed a normal (Gaussian) distribution, which proved not to be the case. Therefore, non-parametric statistical tests were applied. For comparison of multiple items (ANOVAs), a Friedman test was used for paired values and a Kruskal–Wallis test for unpaired ones and, in both cases, the tests were followed by a Dunn’s multiple comparison test to compare the rank of the items. For comparison of two items, a Wilcoxon test was used for paired values and a Kolmogorov–Smirnov test for unpaired ones. Table 2 provides a complete overview of the tests performed for the different experiments. Statistical analyses were done using Prism 6 (GraphPad). All figures were designed with Corel-Draw X8 software.

**Table 2.** Statistical tests performed for behavioral observations and EMG recordings

	Figure	Description	Data structure	Type of test	*p* value
A	3*A*	Comparison between stimulations at cold (4°C), cool (21°C) neutral (25°C), hot (34°C)	Paired, non-parametric	Kruskal–Wallis ANOVA	<0.0001
		Cold vs cool		Dunn’s *post hoc* test	n.s.
		Cold vs neutral		Dunn’s *post hoc* test	<0.0001
		Cold vs hot		Dunn’s *post hoc* test	<0.0001
		Cool vs neutral		Dunn’s *post hoc* test	<0.01
		Cool vs hot		Dunn’s *post hoc* test	<0.01
		Neutral vs hot		Dunn’s *post hoc* test	n.s.
B	3*B*	Comparison between cold stimulations (4°C), cold -5N, and cold -obex	Paired, non-parametric	Kruskal–Wallis ANOVA	0.0411
		Cold vs cold -5N		Dunn’s *post hoc* test	n.s.
		Cold vs cold -obex		Dunn’s *post hoc* test	< 0.05.
		Cold -5N vs cold -obex		Dunn’s *post hoc* test	n.s.
C	4*A*	Comparison between stimulations at cold (4°C), neutral (22°C), hot (45°C), and cold -obex	Paired, non-parametric	Friedman ANOVA	<0.0001
		Cold vs neutral		Dunn’s *post hoc* test	<0.001
		Cold vs hot		Dunn’s *post hoc* test	<0.001
		Cold vs cold -obex		Dunn’s *post hoc* test	n.s.
		Neutral vs hot		Dunn’s *post hoc* test	n.s.
		Neutral vs cold -obex		Dunn’s *post hoc* test	<0.05
		Hot vs cold -obex		Dunn’s *post hoc* test	n.s.
D	N/A	Comparison between responses in Figures 3, 4*A* when different temperatures are used	Non-parametric		
		Neutral 22°C vs neutral 25°C		Kolmogorov–Smirnov *t* test	0.2644
		Hot 34°C vs 45°C		Kolmogorov–Smirnov *t* test	0.0495
		-obex with bath at 25°C vs 22°C		Kolmogorov–Smirnov *t* test	<0.01
E	N/A	Comparison of response rates to cold (4°C) and neutral (22°C) following anesthesia by hypothermia or isoflurane	Non-parametric		
		Cold hypothermia vs isoflurane		Kolmogorov–Smirnov *t* test	0.3077
		Neutral hypothermia vs isoflurane		Kolmogorov–Smirnov *t* test	0.3874
F	4*B*	Comparison between stimulations at cold (4°C), neutral (22°C), hot (45°C), cold -skin, neutral -skin, hot -skin, and cold -obex	Paired, non-parametric	Friedman ANOVA	<0.0001
		Cold vs neutral		Dunn’s *post hoc* test	<0.01
		Cold vs hot		Dunn’s *post hoc* test	<0.01
		Cold vs cold -skin		Dunn’s *post hoc* test	n.s.
		Cold vs neutral -skin		Dunn’s *post hoc* test	<0.0001
		Cold vs hot -skin		Dunn’s *post hoc* test	<0.0001
		Cold vs cold -obex		Dunn’s *post hoc* test	<0.001
		Neutral vs hot		Dunn’s *post hoc* test	n.s.
		Neutral vs cold -skin		Dunn’s *post hoc* test	<0.05
		Neutral vs neutral -skin		Dunn’s *post hoc* test	n.s.
		Neutral vs hot -skin		Dunn’s *post hoc* test	n.s.
		Neutral vs cold -obex		Dunn’s *post hoc* test	n.s.
		Hot vs cold -skin		Dunn’s *post hoc* test	<0.05
		Hot vs neutral -skin		Dunn’s *post hoc* test	n.s.
		Hot vs hot -skin		Dunn’s *post hoc* test	n.s.
		Hot vs cold -obex		Dunn’s *post hoc* test	n.s.
		Cold -skin vs neutral -skin		Dunn’s *post hoc* test	<0.0001
		Cold -skin vs hot -skin		Dunn’s *post hoc* test	<0.001
		Cold -skin vs cold -obex		Dunn’s *post hoc* test	<0.01
		Neutral -skin vs hot -skin		Dunn’s *post hoc* test	n.s.
		Neutral -skin vs cold -obex		Dunn’s *post hoc* test	n.s.

		Hot -skin vs cold -obex		Dunn’s *post hoc* test	n.s.
		Cold vs cold-skin		Wilcoxon *t* test	0.25
		Cold vs cold -obex		Wilcoxon *t* test	0.0010
		Hot vs hot -skin		Wilcoxon *t* test	0.0898
		Neutral vs neutral -skin		Wilcoxon *t* test	0.0078
		Cold -skin vs cold-obex		Wilcoxon *t* test	0.0015
G	6*A*	EMG amplitudes for cold (4°C), neutral (22°C), hot (45°C) cold -5N, neutral -5N, hot -5N, and cold -obex	Unpaired, non-parametric	Kruskal–Wallis ANOVA	<0.0001
		Cold vs neutral		Dunn’s *post hoc* test	<0.0001
		Cold vs hot		Dunn’s *post hoc* test	<0.0001
		Cold vs cold -5N		Dunn’s *post hoc* test	<0.0001
		Cold vs neutral -5N		Dunn’s *post hoc* test	<0.0001
		Cold vs hot-5N		Dunn’s *post hoc* test	<0.0001
		Cold vs cold -obex		Dunn’s *post hoc* test	<0.0001
		Neutral vs hot		Dunn’s *post hoc* test	n.s
		Neutral vs cold -5N		Dunn’s *post hoc* test	<0.0001
		Neutral vs neutral -5N		Dunn’s *post hoc* test	<0.01
		Neutral vs hot -5N		Dunn’s *post hoc* test	n.s.
		Neutral vs cold -obex		Dunn’s *post hoc* test	n.s.
		Hot vs cold -5N		Dunn’s *post hoc* test	<0.001
		Hot vs neutral -5N		Dunn’s *post hoc* test	<0.01
		Hot vs hot -5N		Dunn’s *post hoc* test	n.s.
		Hot vs cold -obex		Dunn’s *post hoc* test	n.s.
		Cold -5N vs neutral -5N		Dunn’s *post hoc* test	<0.0001
		Cold -5N vs hot-5N		Dunn’s *post hoc* test	<0.0001
		Cold -5N vs cold -obex		Dunn’s *post hoc* test	<0.001
		Neutral -5N vs hot -5N		Dunn’s *post hoc* test	n.s.
		Neutral -5N vs cold -obex		Dunn’s *post hoc* test	<0.05
		Hot -5N vs cold -obex		Dunn’s *post hoc* test	n.s.
H	6*B*	EMG amplitudes comparisons for cold (4°C), neutral (22°C), hot (45°C) when all responses are considered and when only responses >0 are considered			
		Cold vs cold >0		Kolmogorov–Smirnov *t* test	0.9998
		Neutral vs neutral >0		Kolmogorov–Smirnov *t* test	<0.0001
		Hot vs hot >0		Kolmogorov–Smirnov *t* test	<0.0001.
I	7	Comparisons of latencies for cold (4°C), neutral (22°C), and hot (45°C)	Unpaired, non-parametric	Kruskal–Wallis ANOVA	<0.0001
		Cold vs neutral		Dunn’s *post hoc* test	<0.0001
		Cold vs hot		Dunn’s *post hoc* test	<0.0001
		Neutral vs hot		Dunn’s *post hoc* test	<0.0001
J	N/A	Comparison of amplitudes after cold (4°C) stimulations for right and left FL	Unpaired, non-parametric	Kolmogorov–Smirnov *t* test	0.1726
K	N/A	Comparison of latencies after cold (4°C) stimulations for right and left FL	Unpaired, non-parametric	Kolmogorov–Smirnov *t* test	0.6001

-5N, trigeminal transection; -obex, complete transection of the spinoencephalic junction, caudal to the obex; -skin, facial skin removal; N/A, non-applicable; n.s., not significant.

## Results

### FLs movements in response to thermal stimulations

**Figure 3.**
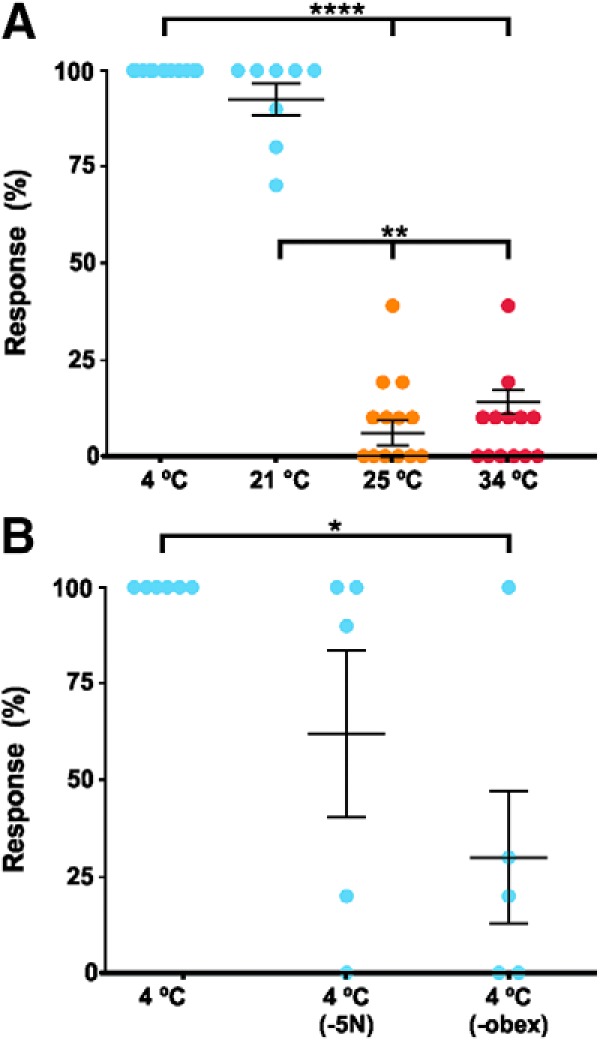
Percentage of FL responses of (***A***) 13 newborn opossums after cold (blue: 4°C, 21°C), neutral (orange: 25°C; bath temperature), or hot (red: 34°C) stimulations; (***B***) five of these specimens were tested for cold (4°C) both before and after trigeminal nerve transection (-5N) and, then, after spinal transection caudal to the obex (-obex). Each dot represents one specimen. Whisker plots stand for mean ± SEM, and thick horizontal lines indicate statistical differences between columns (Extended Data Fig. 3-1*A*,*B*); *p < 0.05, **p < 0.01, ****p < 0.0001.

In a first series of experiments, with bath temperature at 25°C, 13 opossums aged P0–P4 were pinned out to a Sylgard-lined Petri dish with their FLs free to move. The specimens were stimulated by consecutive ejections of liquid at 4°C, 21°C, 25°C (neutral) or 34°C on the muzzle, to observe FL movements under a microscope. The specimens either did not move their FL at all, thus marked as no-response, or moved their FL in an uncoordinated or in a rhythmic fashion (see Materials and Methods). No distinction is made here between uncoordinated and rhythmic movements for the movement response analysis (but see section “Locomotor-like movements of FLs” below). Stimulations at 4°C and 21°C induced a generalized contraction of the axial musculature, as evidenced by rib and pectoral girdle movements, followed by extension of one or both FL in 100.0 ± 0.0% (*n* = 130) and 92.5 ± 4.1% (*n* = 80) of trials, respectively (Fig. 3*A*); Extended Data Fig. 3-1*A*. Similar responses were induced in only 9.2 ± 3.3% and 8.5 ± 3.2% of the trials for stimulations at 25°C and at 34°C, respectively (*n* = 130 in each case). An ANOVA (*p* < 0.0001, Kruskal–Wallis ANOVA; Table 2) with *post hoc* tests comparing these values showed that responses to 4°C and 21°C stimulations differ significantly from those after stimulations at 25°C and 34°C, but not between them. This indicates that newborn opossums are significantly more sensitive to colder than to hotter temperatures, and that even a relatively small difference in temperature (21°C vs 25°C) is enough to induce reliable FL responses. We tested the sensitivity to cold with puff ejections of 10 μl of liquid at 4°C (≤10% of the usual volume) on the facial skin of four specimens, which induced FL movements in 100 ± 0.0% of the trials (Extended Data Fig. 3-1*F*).

10.1523/ENEURO.0347-18.2019.f3-1 Extended Data Figure 3-1 FL response rates for behavioral observation experiments. Download Figure 3-1, DOCX file.

Five of the 13 specimens tested above were subjected to a bilateral transection of the trigeminal nerves and then stimulated with ejections of the 4°C solution, in which case the response rate decreased to 62.0 ± 21.5% (Fig. 3*B*; Extended Data Fig. 3-1*B*). A second transection at the spinoencephalic junction caudal to the obex further lowered the response rate to 30.0 ± 18.4% (*n* = 50). An ANOVA (Kruskal–Wallis ANOVA) with *post hoc* tests comparing all stimulations at 4°C in these five specimens showed a significant difference in the responses only before transection and after complete spinalization (*p* < 0.05; Table 2). These results suggest that cold perception is mediated by cephalic sensory systems, such as the trigeminal nerve. However, since trigeminal transection did not entirely abolish the FL movements, it is possible that cold receptors from the neck or arms were also stimulated.

The tail and hindlimbs were stimulated by ejections of cold solution, before and after transections, which nearly always induced FL movements (data not shown). These responses were not quantified. Nonetheless, because cold stimulations of these body parts were very potent at inducing motor responses, they routinely served to verify the responsiveness of the preparations, especially after nervous tissue sections or skin removal.

**Figure 4.**
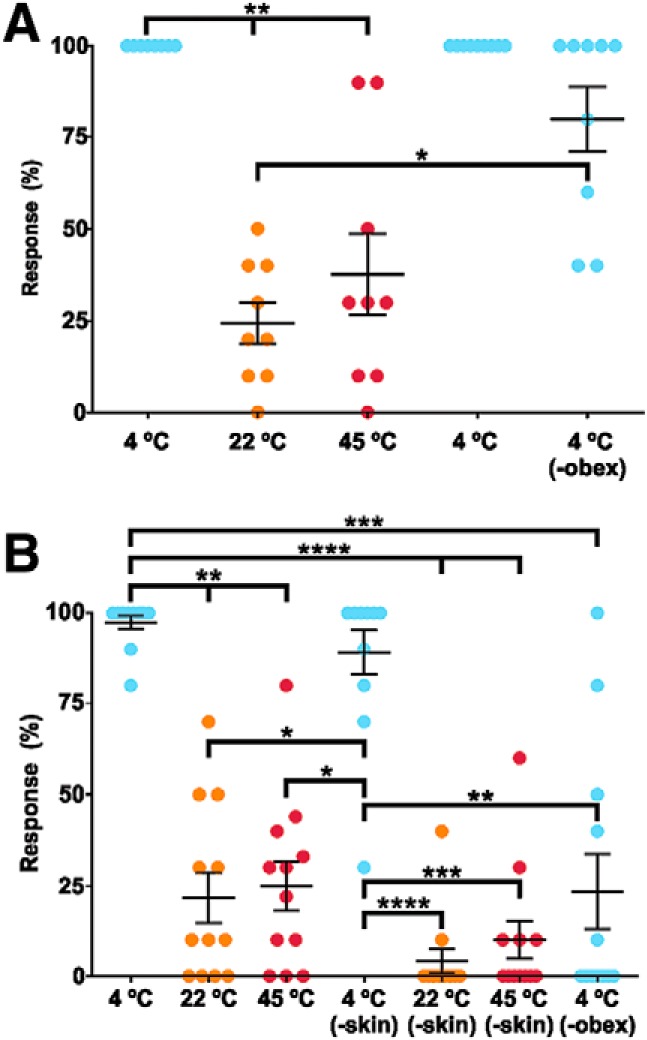
Percentage of FL responses after cold (blue: 4°C), neutral (orange: 22°C; bath temperature) or hot (red: 45°C) stimulations (***A***) before and after transection of the spinal cord caudal to the obex (-obex) alone, or (***B***) after facial skin removal (-skin) followed by spinal transection. Each panel represents a series of experiments during which the specimens were consecutively stimulated. In both panels, each dot represents one specimen and whisker plots stand for mean ± SEM, and thick horizontal lines indicate statistical differences between columns (Extended Data Fig. 3-1*C*,*D*); *p < 0.05, **p < 0.01, ***p < 0.001, ****p < 0.0001.

In a second series of experiments, with bath temperature at 22°C, nine different specimens were stimulated as before at 4°C and 22°C (neutral) temperature, and then with a solution at 45°C (Fig. 4*A*; Extended Data Fig. 3-1*C*). As expected, cold stimulations induced FL movements in 100.0 ± 0.0% of the trials. Neutral and hot stimulations were effective in 24.4 ± 5.6% and 37.8 ± 11.0% of the trials, respectively. An ANOVA with *post hoc* tests showed that responses to cold differ statistically from responses to neutral and hot stimulations (*p* < 0.0001, Friedman ANOVA; Table 2). After another series of cold stimulations, which still elicited responses in 100.0 ± 0.0% of the trials, a complete transection at the obex decreased the response rate to cold stimulations to 80.0 ± 8.8%.

It must be noted that the response rates obtained following 22°C and 45°C stimulations were ∼2.7× and 4.4× those recorded in the previous series of experiments for stimulations at 25°C and at 34°C, respectively, whereas the response rates to 4°C stimulation after section at the obex is 2.7× that recorded in the previous series of experiments in the same condition; *t* tests (Kolmogorov–Smirnov) used to compare the response rates between the two series of experiments revealed no statistical difference for 22°C versus 25°C (*p* = 0.2644), a slight difference for 34°C versus 45°C (*p* = 0.0495), and a larger one for the response rates to 4°C after section (*p* < 0.01; Table 2). These observations, especially the last one, suggest that the higher response rate recorded for 45°C may not be due entirely to the warmer temperature but also to higher reactivity of the specimens used in the second series of experiments.

It has been reported in adult mammals that a first exposure to noxious cold may induce hypersensitity to further cold exposure (Beise et al., 1998; Khasabov et al., 2001). Hypothermia was used to anaesthetize specimens and this may have affected the response to cold recorded *in vitro*. To test this hypothesis, four opossums (P1) were deeply anaesthetized with isoflurane and processed for *in vitro* stimulations as previously described. Stimulation with ejection of liquid at 4°C and 22°C (neutral) triggered FL movements in 97.5 ± 2.5% and 12.5 ± 4.8% of the trials, respectively (Extended Data Fig. 3-1*E*); *t* tests (Kolmogorov–Smirnov) used to compare the response rates in specimens anesthetized by hypothermia to those anesthetized by isoflurane showed no statistical difference (*p* = 0.3077 for 4°C; *p* = 0.3874 for 22°C; Table 2). Hypothermia used as an anesthetizing method does not seem to affect the sensitivity of newborn opossums to cold stimulation.

In an attempt to determine the localization of the cold receptors, another group of 12 specimens anesthetized by hypothermia was used. Initial response rate was recorded after stimulation with cold (4°C), neutral (22°C), and hot (45°C) liquid, which induced responses in 97.5 ± 1.8%, 21.7 ± 6.9%, and 24.9 ± 6.8% of the trials, respectively (Fig. 4*B*; Extended Data Fig. 3-1*D*). The skin covering the head was then removed, and the preparations were stimulated again, eliciting response rates of 89.2 ± 6.1% for cold, 4.2 ± 3.4% for neutral, and 10.0 ± 5.2% for hot (Fig. 4*B*; Extended Data Fig. 3-1*D*). ANOVA with *post hoc* tests (*p* < 0.0001, Friedman ANOVA; Table 2) revealed that response rates to cold, both with and without skin, were significantly different from response rates recorded in all other conditions of stimulation; *t* tests (Wilcoxon; Table 2) aimed at comparing the response rates before and after skin removal for each temperature showed that the decrease of response to cold was not significant (*p* = 0.25), the decrease of response to hot was also not significant (*p* = 0.0898), whereas the decrease for the neutral temperature stimulations was statistically significant (*p* < 0.01). Skin removal having barely decreased the FL responses to cold, it is possible that the receptors activated by cold are located in the remaining tissues of the dermis, the trigeminal ganglia or the neuraxis. Therefore, in four specimens, we tested whether puff ejections (10 per specimen) of cold liquid directly inside the skull, toward the brainstem and trigeminal ganglia (5G), induced motor responses; it did in only 5.0 ± 3.5% of the trials (*n* = 40 stimulations; Extended Data Fig. 3-1*G*). Then a complete transection was performed at the caudal obex in the 12 skin-free specimens, which were then stimulated with cold solution. This induced FL movements in 23.3 ± 10.3% of the trials (Fig. 4*B*; Extended Data Fig. 3-1*D*), a significant decrease compared to the responses to cold recorded before (*p* = 0.001; Wilcoxon *t* test; Table 2) or even after skin removal before the transection (*p* = 0.0015; Wilcoxon *t* test; Table 2). These data suggest that cold thermoreceptors are located in the tissues underlying the skin but not in the brainstem or the 5G.

### Locomotor-like movements of FLs

**Figure 5.**
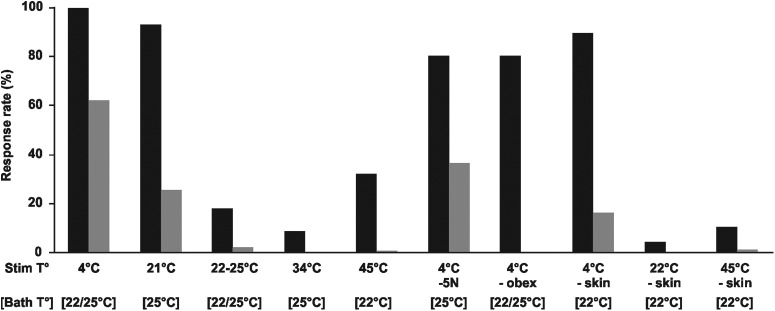
Response rates for all responses (“uncoordinated + rhythmic”; black columns) and rhythmic responses only (gray columns) after thermal stimulations (Stim T°) for all FL movements experiments (Extended Data Fig. 4-1). The neutral temperatures ([Bath T°]) are given for all cases. -5N, transection of the trigeminal nerve; -obex, transection of the neuraxis, caudally to the obex; -skin, removal of facial skin.

In all series of experiments described previously, stimulations by liquid ejections sometimes induced rhythmic movements of the FL, in which case an initial extension of both FL was observed, followed by alternate extensions of the left and right FL (Fig. 1*B*, lower rows; Movie 3). This pattern of alternation is similar to that performed by newborn opossums during locomotion (Pflieger et al., 1996; VandeBerg and Williams-Blangero, 2010). We analyzed the frequency of these rhythmic movements in preparations that had not been sectioned nor flayed on the face. Cold stimulations induced rhythmic responses in 61.8% of trials at 4°C (*n* = 272/440 stimulations) and in 25% of the trials at 21°C (*n* = 20/80; bath at 25°C), but only in 1.7% of the trials at neutral (bath temperature at either 22°C or 25°C; *n* = 6/350) temperatures (Fig. 5; Extended Data Fig. 4-1). Stimulations at 34 and 45°C were even less effective as they induced rhythmic movements in 0.0% (*n* = 0/130) and 0.5% (*n* = 1/217) of the trials, respectively. After transection of the trigeminal nerves, stimulations at 4°C induced rhythmic movements in 36.0% of trials (*n* = 18/50), which were totally abolished after transection caudal to the obex (0.0%, *n* = 0/90). The 12 specimens tested after skin removal performed rhythmic movements in 15.8% of the trials (*n* = 19/120) when stimulated at 4°C, in 0.0% of the trials at neutral temperature (*n* = 0/120), and in 0.8% of the trials at 45°C (*n* = 1/120). The four specimens tested with ejections of 10 μl of cold solution (4°C; ≤10% of the usual volume) directed toward the facial skin showed rhythmic activity in 20.0% of the trial (*n* = 8/40).

10.1523/ENEURO.0347-18.2019.f4-1 Extended Data Figure 4-1 FL responses - either all responses (uncoordinated + rhythmic) or rhythmic responses only - induced by temperature in 34 *in vitro* preparations of newborn opossums in proportion of total stimulations. Download Figure 4-1, DOCX file.

### EMG recordings

To further investigate FL responses induced by liquids at different temperature, we recorded the activity of the triceps brachii muscles in additional *in vitro* preparations of newborn opossums in which the FLs were pinned to the substrate. To compare the EMG amplitude of left and right triceps from different preparations, the amplitude of a given triceps response was first expressed as a percentage of the maximal amplitude obtained for this muscle (see Materials and Methods). The normalized values of all recordings were then averaged (±SEM). Except when specified otherwise, all the data obtained were pooled for analysis, including the absence of response (EMG amplitude of 0%).

**Figure 6.**
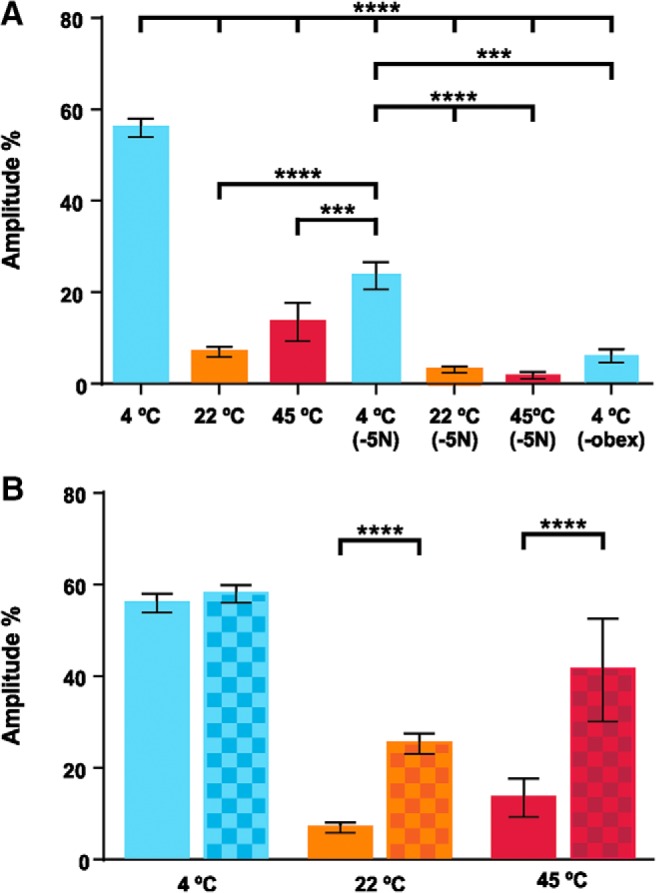
EMG recordings of the triceps muscles following thermal stimulations. ***A***, Response amplitudes to cold (blue: 4°C) or neutral (orange: 22°C; bath temperature), and hot (red: 45°C) temperatures before and after trigeminal nerve transection (-5N) and, then, after spinal transection caudal to the obex (-obex). The amplitude given represents the average of individual muscle responses that were normalized to the highest response amplitude for that muscle during the series of experiments. ***B***, EMG amplitude of responses to cold, neutral, and hot temperature before (plain columns) and after (checkered columns) no-responses (amplitudes = 0) were removed from the analysis. In all panels, whisker plots stand for mean ± SEM, and thick horizontal lines indicate statistical differences between columns (Extended Data Fig. 5-1*A*,*B*); ***p < 0.001, ****p < 0.0001.

Thirteen specimens were stimulated with consecutive ejections of cold (4°C), neutral (22°C), and hot (45°C) solutions separated by a 40 s interval. EMG response amplitudes were 56.6 ± 2.0% (*n* = 21 limbs, 201 stimulations), 5.8 ± 0.8% (*n* = 21 limbs, 290 stimulations), and 13.5 ± 4.2% (*n* = 9 limbs, 89 stimulations), respectively, for cold, neutral, and hot temperature stimulations (Fig. 6*A*; Extended Data Fig. 5-1*A*). Keeping the electrodes in place, the specimens were transferred under a dissecting microscope to perform a complete and bilateral transection of the trigeminal nerves, before being returned to the recording platform to be stimulated again. Following cold stimulations, response amplitudes decreased to 23.7 ± 3.0% (*n* = 10 limbs, 100 stimulations), and those to neutral (0.3 ± 0.2%; *n* = 17 limbs, 170 stimulations) and hot (1.8 ± 0.8%; *n* = 7 limbs, 70 stimulations) stimulations were virtually abolished. A complete transection of the neuraxis caudal to the obex was then performed, which led to a further decrease of response amplitude to 6.3 ± 1.5% in response to cold stimulations (*n* = 10 limbs, 88 stimulations). An ANOVA (Kruskal–Wallis with *post hoc* tests) shows that response amplitudes to cold stimulation before any section are significantly higher than response amplitudes recorded in all other conditions (*p* < 0.0001; Table 2). Moreover, response amplitudes to cold stimulation after section of the trigeminal nerves are higher than those to neutral (*p* < 0.0001) and those to hot (*p* < 0.01) before sections.

10.1523/ENEURO.0347-18.2019.f5-1 Extended Data Figure 5-1 Amplitude of EMG responses to different temperatures. Download Figure 5-1, DOCX file.

For all EMG experiments before sectioning, the ratio of responses (amplitude > 0)/stimulations for cold, neutral and hot was, respectively, 96.5% (*n* = 194/201 stimulations), 23.1% (*n* = 67/290), and 32.6% (*n* = 29/89). The low occurrence of responses to neutral and hot stimulations implies that a large number of null responses (amplitude = 0) were used to compute the amplitudes given previously. We therefore computed the amplitude obtained before trigeminal sections without the null responses and found EMG amplitudes of 58.7 ± 1.9%, 25.2 ± 2.1%, and 41.4 ± 11.2% following cold, neutral and hot stimulations, respectively (Fig. 6*B*; Extended Data Fig. 5-1*B*). When compared to the results comprising the null responses, the differences in amplitude are statistically significant for neutral and hot stimulations (*p* < 0.0001, Kolmogorov–Smirnov *t* tests), but not for cold stimulations (*p* = 0.9998, Kolmogorov–Smirnov *t* tests) (Table 2). These results indicate that, when they occur, the responses to neutral temperature have an average amplitude corresponding to 49.4% of the amplitude of responses to cold, and the responses to hot temperature have an average amplitude of 78.3% that of responses to cold stimulation.

**Figure 7.**
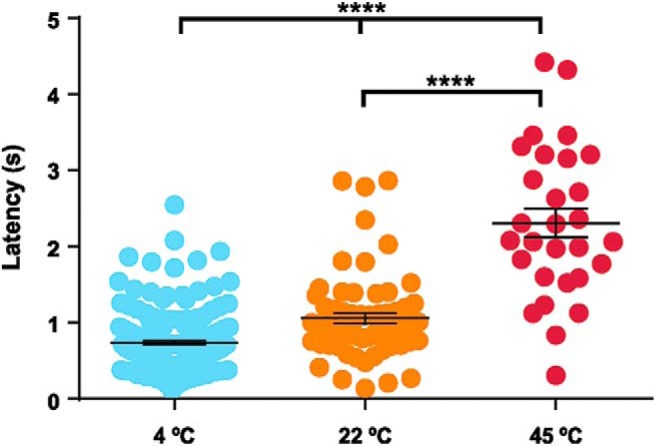
Latencies of EMG responses after cold, neutral, and hot stimulations; each dot represents one triceps muscle response. In all panels, whisker plots stand for mean ± SEM, and thick horizontal lines indicate statistical differences between columns (Extended Data Fig. 6-1*A*); ****p < 0.0001.

The latencies of responses were also measured on EMG responses recorded before transection. Latency was the shortest following stimulation with cold liquid, at 741 ± 27 ms (*n* = 194 stimulations), the longest after hot stimulations, at 2303 ± 185 ms (*n* = 29), and intermediate after neutral stimulations at 1059 ± 68 ms (*n* = 67; Fig. 7; Extended Data Fig. 6-1*A*). All differ significantly from each other (*p* < 0.0001, Kruskal–Wallis ANOVA; Table 2).

10.1523/ENEURO.0347-18.2019.f6-1 Extended Data Figure 6-1 Latency of FL responses after temperature stimulations for EMG recordings. Download Figure 6-1, DOCX file.

The EMG were successfully recorded in both left and right triceps in nine of these 13 preparations. Interlimb comparisons were performed only on the responses to cold stimulations (before section) since they occurred more frequently and with greater amplitude. The response amplitudes averaged 53.4 ± 3.5% for the right triceps and 61.2 ± 3.2% for the left triceps (*n* = 69 stimulations for each pairs of limbs; Extended Data Fig. 5-1*C*), a difference that is not statistically significant (*p* = 0.1726, Kolmogorov–Smirnov *t* test; Table 2). The latencies of 842 ± 57 and 725 ± 38 ms for the right and left triceps, respectively (*n* = 69; Extended Data Fig. 6-1*B*) were also not statistically different (*p* = 0.6001, Kolmogorov–Smirnov *t* test, Table 2). Following cold stimulations (4°C), two specimens responded by a synchronous burst of the left and right triceps, which then switched to alternate right and left bursts (Fig. 2*E*, magenta lines). Similar responses were observed 70% of the times in one of the specimens, and 20% of the time in the other. In contrast to the preparations in which the FLs were free to move and alternate movements were often observed, the FL were pinned to the substrate for the EMG recordings, which may have prevented the expression of left-right alternation by reducing proprioceptive feedback.

### TRPM8 immunohistochemistry and RT-PCR

**Figure 8.**
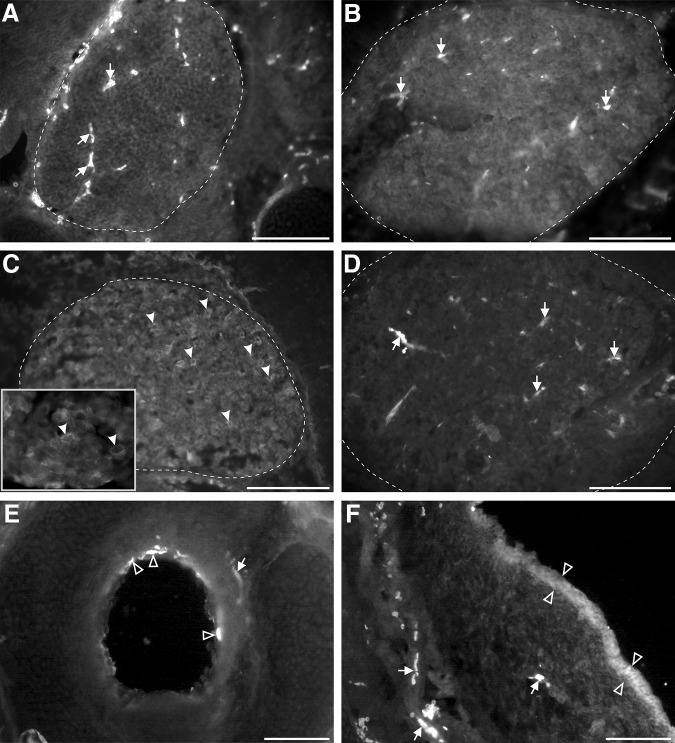
TRPM8 immunoreactivity in transverse sections of cephalic tissues of newborn opossums. ***A–D***, Trigeminal ganglia (approximately delineated by a dashed line) at P1 (***A***), P9 (***B***), and P13 (***C***, ***D***) processed with (***A–C***) or without (***D***) the primary antibody against TRPM8. Labeled cell bodies are present only at P13 (examples pointed by arrowheads in ***C***). The inset in C shows some labeled cell bodies at higher magnification. ***E***, Labeled apical membrane of epithelial cells (empty arrowheads) in the trachea of a P9 opossum. ***F***, Snout from a P9 opossum showing diffuse TRPM8 labeling in the epidermis (between empty arrowheads). Arrows in ***A***, ***B***, ***D***–***F*** point to blood vessels intrinsically fluorescent. Scale bar in ***F*** = 100 μm (for ***A–F***).

The previous results show that cold is a potent stimulus to induce FL motor responses in newborn opossums, in contrast to neutral and hot. In most mammalian species studied, TRPM8 is a crucial cold thermoreceptor (Schepers and Ringkamp, 2010; Vriens et al., 2014). We thus sought to ascertain its expression in newborn opossums using immunohistochemistry. At P0, TRPM8 labeling was detected at the apex of a few epithelial cells dispersed in the aerial pathways, especially in the trachea, of all specimens (Fig. 8*E*, empty arrowheads), but not in the central nervous system, sensory ganglia, or nerves (Fig. 8*A*). We processed tissues from older opossums, and saw the same pattern of labeling at P6, P7, and P9 (Fig. 8*B*). TRPM8 labeling was detected in the trigeminal ganglia of only two of the five P13–P14 specimens (Fig. 8*C*, filled arrowheads; compare to control sections processed without the primary antibodies, Fig. 8*D*). At all ages studied, a few sections from some specimens showed diffuse patches of putative TRPM8 labeling within the epidermis (Fig. 8*F*, empty arrowheads).

**Figure 9.**
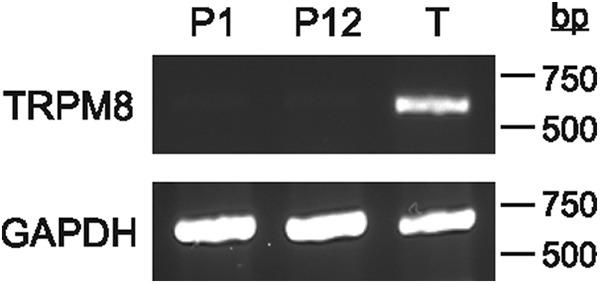
RT-PCR gels of GAPDH and TRPM8 in the head of young opossums (P1, P12) and in the testes of an adult (T), illustrating the absence of TRPM8 mRNA expression at young ages.

To verify these findings, we assessed the presence of TRPM8 mRNA in head tissues of P1–P12 opossums by RT-PCR. Adult testes mRNA was used as a positive control for TRPM8 primers. GAPDH amplification from all samples shows that total RNA was successfully extracted and reverse-transcribed. However, no amplification of TRPM8 mRNA was obtained from head tissues, whereas it was from the testes (Fig. 9). Together, immunohistochemical and RT-PCR findings suggest that TRPM8 is not the receptor involved in the sensorimotor response to cold stimulation in newborn opossums.

## Discussion

To study the sensitivity of neonatal opossums to external temperature, we observed FL movements and triceps muscle contractions induced by thermal stimulations of the snout in *in vitro* preparations in which most peripheral and central networks components were preserved. We found that cold temperatures proved far more potent than warm temperatures to induce FL responses. Indeed, in all series of experiments, cold stimulations systematically induced strong FL responses, whereas neutral and hot stimulations elicited less frequent responses and of lesser intensities and of longer latencies. Sectioning of the trigeminal nerves and removing the skin nearly abolished responses to neutral and hot stimulations, but only decreased responses to cold stimulations. Even after a section just caudal to the obex, cold stimulations still induced sporadic FL responses. These residual responses are most likely due to the liquid diffusing to portions of the skin or underlying tissues innervated by other cranial nerves, such as the vagus, or by spinal nerves. Direct ejections of cold liquid on the neuraxis or on trigeminal ganglia did not induce FL motor responses, supporting the idea that temperature is indeed perceived by peripheral sensory fibers.

FL responses to cold ejection were still strong after facial skin removal. Even if cold liquid may have stimulated receptors on the skin of the neck or FL, explaining part of the responses, cold receptors may still be present in the remaining facial tissues. The dermis of the facial skin is poorly developed in newborn opossums, but contains numerous nerve fibers (Jones and Munger, 1985; Adadja et al., 2013). The skin is glabrous and no dermal muscles are observed, the tongue and masticatory muscles are proportionally well developed and innervated. Moreover TRPM8 expression has not be found in the latter in the mature rat (Yajima et al., 2015). Facial thermoreceptors activated after skin removal may be from nerve fibers in the remaining dermis, injured or not, or from sensory cells of the nasal and oral mucosae now exposed to the stimulation.

The possibility of cold liquid spreading to the caudal part of the head and to the body also explains the variable results recorded following transection caudal to the obex and prevents us from identifying specific pathways relaying cold thermosensory inputs to the spinal cord. Nonetheless, our results allow some general inferences. First, the trigeminal system plays a role in these responses as sections of the 5N decreased both the response rates of the FL and the triceps EMG amplitudes. Second, other cranial nerves convey cold sensation in newborn opossums as shown by the further decrease in response rates and EMG amplitudes when a complete transection of the spinoencephalic junction is performed after 5N transection. Third, part of the response is mediated by descending pathways as suggested by the strong decrease in EMGs amplitudes for cold stimulations after spinoencephalic transection, in specimens for which less skin was left on the FL and neck. Fourth, projections descending from the brain are important to generate rhythmic, locomotor-like responses of the FL as these responses persisted after 5N sections or skin removal but were abolished after spinoencephalic transections.

Concerning the last point, the effect of the transection may be due to a general decrease in the sensory drive impinging on the spinal circuitry generating limb movements or to the section of specific pathways necessary to induce rhythmic activity. As for the latter, the most probable candidates are reticulospinal projections, which form the bulk of descending projections in newborn opossums (Wang et al., 1992). Moreover, the reticulospinal system is a crucial component of the supraspinal control of locomotion in vertebrates (for review, see Grillner, 2003; Rossignol et al., 2006; Brownstone and Chopek, 2018).

The motor responses obtained following stimulation to neutral temperature may seem surprising, but could be attributed to stimulation of skin mechanoreceptors induced by liquid movement. In similar *in vitro* preparations of newborn opossums, facial pressures induced triceps muscle contractions (Desmarais et al., 2016). These contractions were decreased, but not totally abolished (5% of the maximal amplitude), after skin removal, possibly due to exposure of free nerve endings in the remaining dermis. The fact that responses to neutral temperature stimulations herein were nearly abolished by facial skin removal or 5N transections supports this interpretation. However, mechanosensation, if involved, likely explains part of the responses to any temperature. By comparison to responses to neutral stimulations, triceps responses following cold stimulations were on average 4–5× more frequent, with latencies 300 ms shorter and amplitudes 4–5× higher. This clearly supports that cold stimulation solicited cold thermoreceptors in addition to possible mechanosensory components due to pressure of the liquid ejection.

A study on the coding of temperature by spinal dorsal horn neurons in the adult mouse revealed that the amplitude of responses to cold is correlated to ΔT°, whereas responses to heat are correlated with the absolute value of the target temperature (i.e., warmer stimulating temperatures generate large responses even with a low ΔT°; Ran et al., 2016). By contrast, in newborn opossums, a deviation from the neutral temperature, either small or large, seems sufficient to induce strong FL responses to cold. Indeed, with the bath at 25°C, stimulations at 21°C induced FL movements at rates comparable to stimulations at 4°C (92.5% vs 100%), while stimulations at 22°C were not as effective to induce FL responses when the bath was at 22°C (21.4%–24%). Concerning the responses to hot temperature, our results show a relationship with increased temperature but do not permit to distinguish whether responses are more dependent on the ΔT° or the absolute value since stimulations at 34°C induced a response rate of 8.5% when the bath was at 25°C (ΔT° = +9°C) and stimulation at 45°C induced response rates of 24.9%–37.8% when the bath was at 22°C (ΔT° = +23°C).

However, the effect of hot temperature stimulations is complex. Indeed, when all responses to stimulation, including their absence (i.e., amplitude = 0), are considered, the results do not differ significantly from those obtained after neutral stimulations, which would suggest that mechanosensation explains the responses. However, when only the responses with an amplitude >0 are considered in the analysis, latencies of responses to hot stimulations are about twice that of neutral stimulations (2.3 vs 1.1 s, respectively) and their variability is about thrice that of neutral stimulations (SEM of 184.8 vs 68.1 ms, respectively). Also, amplitudes of responses to hot stimulations are on average 1.7× that of responses to neutral stimulations (41.4% of maximal response vs 25%, respectively), and their variability is also greater (SEM of 11.2% vs 4.2%, respectively, for hot and neutral). Thus, it is possible that thermoreceptors, in addition to mechanoceptors, are affected by hot stimulations. The larger variability of responses to hot stimulations could be interpreted by activation of central inhibitory circuits in addition to excitatory ones. A mixture of inhibitory and excitatory inputs would result in a larger variability in the frequency, amplitude and latency of responses to hot stimulations. In immature networks inhibitory neurotransmitters (glycine, GABA) often exert an excitatory effect on neurons, depending on the chloride homeostasis mechanisms of the latter (for review, see Vinay and Jean-Xavier, 2008; Blaesse et al., 2009; Ben-Ari et al., 2012). It is generally accepted that the potassium-chloride cotransporter 2 (KCC2), that extrudes chloride from cells, and the sodium-KCC1 (NKCC1), that accumulates it, play a major role in the regulation of chloride. During neuron development, KCC2 becomes more expressed or efficient and NKCC1 less so, resulting in a gradual switch from a depolarizing to a hyperpolarizing response to inhibitory neurotransmitters. For example, in *in vitro* preparations of rats aged E16 to P6, trigeminal nerve stimulations point to an excitatory action of GABA in neurons of the principal trigeminal nuclei, an effect peaking around E20 and P1 (Waite et al., 2000). An immunohistochemical study of the distribution of different proteins linked to the GABA physiology, glutamic acid decarboxylase, vesicular GABA transporter, KCC2, in the interpolaris part of the spinal trigeminal nucleus in embryonic mice led Kin et al. (2014) to suggest that the switch occurs between E13 and E17 in this species. The expression of KCC2 and NKCC1 in the opossum’s spinal cord indicates that the development of inhibition in this species is broadly comparable to that in rodents (Phan and Pflieger, 2013). It is thus possible that, at the ages studied here, P0–P4 opossums, which compares to E11.5–E17.5 rodents, inhibitory neurotransmitters exert a mixed action, sometimes excitatory and sometimes inhibitory. In that case, the variability of responses recorded for hot stimulation may reflect the central activation of both excitatory and mature inhibitory (i.e., physiologically inhibitory) components by afferents sensible to warmer temperatures. By contrast, the higher frequencies of occurrence and larger amplitudes of responses following cold stimulations suggest that cold afferents activate mainly excitatory or immature inhibitory circuits (i.e., physiologically excitatory), at the ages studied.

That innocuous warm temperature has inhibitory or suppressing effects on motor behaviors in young marsupials and that this effect could be linked to maturation, is supported by the following observations on Tammar wallabies (*Macropus eugenii*) aged from P15 and over (Ho, 1997). Animals were removed from the mother’s pouch and laid supine on a holder to induce FL locomotion. When the ambient temperature was increased from 25°C–37°C in <5 min the frequency of the ongoing locomotor rhythm decreased to ∼70% of the initial value at younger ages (P15–P39) and halted at older ages (≥P40). At all ages, a return to a temperature of 25°C stimulated FL locomotor activity, supporting the idea that external temperatures influence this behavior. However, Nicholls et al. (1990) reported that in *in vitro* preparations of isolated brainstem-spinal-cord of P0–P3 opossums (*M. domestica*), both the amplitude of reflex responses recorded in ventral roots and the frequency of spontaneous activity were greater at ∼23°C than at ∼28°C. All peripheral receptors having been removed during dissection in their preparations, it is possible that some mechanisms intrinsic to the central nervous system may have depressed motor responses to warmer temperatures.

TRPM8 receptors are activated around 27°C, and their activity increases on cooling until it reaches a plateau around 15°C (McKemy et al., 2002; Peier et al., 2002a), which is inside the thermal range used in our experiments. However, they were not detected in sensory neuron somas and fibers before P13 in the opossums. TRPM8 labeling was however noted in a small number of cells sparsely distributed in the aerial epithelia as early as P1, which supports the specificity of the antibodies for this receptor. Cells in the nasal and oral mucosae of adult rodents express TRPM8 (Abe et al., 2005; Liu et al., 2015). The absence of amplification of TRPM8 in samples from opossums younger than P12 may be explained by the scarcity of labeled cells and the fact that only heads without the trachea were processed for RT-PCR. Putative TRPM8 labeling was also observed as a diffuse background in patches of the epidermis in a few sections, which could be due to truncated epidermal TRPM8 (eTRPM8), an isoform of TRPM8 present in the endoplasmic reticulum of keratinocytes that plays a cold-dependent role in the proliferation and differentiation of these cells (Denda et al., 2010; Bidaux et al., 2015, 2016). eTRPM8 would not have been amplified by the primers used herein for TRPM8.

Based on physiologic recordings of dissociated spinal DRG cells and gene expression experiments, Hjerling-Leffler et al. (2007) proposed a model of sequential emergence of some thermoreceptors in mice, according to which capsaicin-sensitive heat receptors TRPV1 are expressed first, at E11.5–E12.5, followed by menthol-sensitive cold receptors TRPM8, at E16.5. However, they could record DRG neuron responses to cold as early as E11.5 which suggest that receptors other than TRPM8 mediated the responses at this early age. It has been shown in adult rats and mice as well as in chickens that a subpopulation of cold responding sensory neurons is insensitive to menthol (Thut et al., 2003; Babes et al., 2004, 2006; Munns et al., 2007; Yamamoto et al., 2016). It may be the same in newborn opossums where responses to cold are observed before TRPM8 expression. A candidate for TRPM8-independent cold responses could be TRPA1 that is activated by cold temperatures in the noxious range (<17°C) (Story et al., 2003). However, TRPA1 expression appears only after birth in mouse DRG neurons (Hjerling-Leffler et al., 2007) and its ability to transduce cold perception *in vivo* has been questioned (Bandell et al., 2004; Bautista et al., 2006; Pogorzala et al., 2013). Another candidate is TREK-1, a two-pore domain K^+^ channel that closes when temperature decreases, which is abundantly expressed in nervous and non-nervous tissues of mouse embryos (Maingret et al., 2000; Aller and Wisden, 2008). Some sodium channels may also be candidates, such as the epithelial sodium channel (ENaC), which shows amplified sodium currents at temperatures below 37°C and which pharmacological blockade diminishes responses to cold in DRG neurons (Askwith et al., 2001; Thut et al., 2003), or the voltage-gated sodium channels Nav1.6 or Nav1.8, which have been involved in cold-induced pain in adult mice (Zimmermann et al., 2007; Deuis et al., 2013). If such cold-activated receptors and channels are present on sensory nerve endings, the thin skin of newborn opossums (Jones and Munger, 1985) exposed them to variations of external temperatures. During development, the exposure of these nerve endings to external conditions would be reduced by the thickening of the skin. Moreover, the concomitant maturation of TRPM8-expressing epidermal and subepidermal nerve fibers or Merkel cells, as these epidermal mechanosensors may also express TRPM8 (Cahusac and Noyce, 2007; Bouvier et al., 2018), would increase the importance of this receptor in cold thermosensation.

The heat and capsaicin receptor TRPV1 is activated by temperatures >43°C (Caterina et al., 1997). It is expressed from E12.5–E13.5 onwards in mouse DRG (Tamura et al., 2005; Funakoshi et al., 2006; Hjerling-Leffler et al., 2007) and synapses between TRPV1 expressing neurons located in DRG and spinal neurons are thought to be already fully functional at birth in rats (Baccei et al., 2003). It is possible that TRPV1 is similarly expressed and functional in the newborn opossums and account for the responses to hot temperatures observed. However, no TRPV1 homolog has yet been annotated in marsupial genomes, making further investigation of its expression difficult in opossums.

In 1928, Langworthy observed that newborn Virginian opossums (*Didelphis virginiana*) crawl toward a warm source and away from cold when removed from the mother’s pouch (Langworthy, 1928). In another marsupial, the northern quoll (*Dasyurus hallucatus*), a temperature gradient ranging from 29°C to 31°C was observed between the urogenital sinus and the pouch on the female’s belly during gestation only, which led the authors to hypothesize that it may serve to guide the pups toward the pouch at parturition (Nelson and Gemmell, 2005). The results of the present study suggest that, in newborn *Monodelphis* opossums, which is pouch-less, this behavior is a cold avoidance behavior rather than heat tropism: cold stimulates motor activity until competing stimuli, physical or chemical, depress it.

## Synthesis

Reviewing Editor: Muriel Thoby-Brisson, CNRS UMR 5287 Université Bordeaux Decisions are customarily a result of the Reviewing Editor and the peer reviewers coming together and discussing their recommendations until a consensus is reached. When revisions are invited, a fact-based synthesis statement explaining their decision and outlining what is needed to prepare a revision will be listed below. The following reviewer(s) agreed to reveal their identity: Didier Le Ray.

Dear Authors,

Below are the detailed comments of the two reviewers who examined your work. It seems that they both find the need for providing more detailed rationale for different aspects of the study such as: the rationale for the different temperatures used, what is known about thermosensation so far, why has this study been performed?, more consistencies in numbers given in the result/materials/figures legend sections, more explanation on the type of movement examined...... In addition the reviewers suggest new sets of experiments in order 1) to avoid a possible activation of mechanoreceptors and 2) to use another way to anesthetize the animals, hypothermia possibly inducing hypersensibility after exposure to cold temperature. To summarize the study should be much more specifically introduced, the results should be presented in a more rational fashion and the discussion should be extended towards directly connected points (pathway involving trigeminal afferents, TRPM receptor locations...).

Reviewer 1:

General Comments

The opossum preparation provides a unique opportunity to examine the development of neural circuits. This is because they mature outside the mom at developmental stages were mice and rats are in utero. This provides an opportunity to examine the development of networks in unique ways. That said it seems as if the manuscript needs to be revised and some new experiments performed to provide support for the authors conclusions.

Major Comments

For this paper we need more rationale for the temperatures chosen in the introduction.

Experiments need to be performed or literature cited regarding the physiological skin temperature and core body temperature of the opossum. Do opossum joeys show lower physiological core temperatures like perinatal mice do? If so then the rationale of operating in vitro at 20-22C may be more justified. Interpretation of the results is made more difficult without this information.

Possible mechanoreceptor activation due to the force of liquid could be avoided if a split-bath was employed and the liquid heated adjacent to the snout. Alternatively, a small acsf ice cube could be placed close to the snout. Laser evoked heating of the snout could be also employed.

Minor Comments

Page 1. Sp. Keywords.

Significance. Still not quite clear what the general importance is. Lower temperatures induce movement but perhaps should say why this might be important to the newborn animal.

Introduction. Should mention the temperature range of TRPM8 and sensitivity to menthol.

Introduction Page 3. Third paragraphs. Should address the major gap in the literature. Why would the pathways need to be functional?

Page 3. Should also discuss the age range equivalent to mice in more detail. The authors use P0-P14 here. What are the equivalents to mice/rats across this range?

Page 5 perfuse - superfuse.

Page 6. ‘The specimens were pinned to the bath substrate’. There is no mention that this is in vitro. It is assumed from the previous paragraph. I would add to the start of the in vitro preparation section that all experiments were conducted on in vitro animals (assuming this is the case) to avoid any confusion.

Page 6 - Why was the bath kept at 22C? What would be the physiological core temperature of the neonates at the age used and the skin temperature? This could be important for the interpretation of results.

Page 6. The specimen was stimulated 10 times. Why? What is the rationale for choosing 40-120 s intervals? Did habituation of the responses occur?

Page 7 - To prevent aliasing the filter setting presumably needed to be moved to 50 Hz or a heavy smoothing function applied. Can you please clarify or reanalyze with low-pass filters in place. Also, express 0.1 kHz as 100 Hz.

Page 10. The phrase ‘newborn opossums’ is vague. Put age instead (P0/1 presumably).

Page 10. To continue to harp on this point which I think is critical the authors need to carefully rationalize the use of the different temperatures. For example, TRPM8 is thought to be preferentially active between 15-27C. What ThermoTRPs would the authors be activating at the different temperatures chosen and why?

Page 11. Presumably, the solution at 45C was chosen to also recruit TRPV1 but this rationale is not stated explicitly.

Page 13. Stimulation in intact preparations - this is a little confusing. I usually think of intact preparations as non-dissected preparations. I'd reword this.

Page 13. It is interesting that the cold temperature produced movements in 16% of animals with the skin removed. Would this still occur with afferents cut?

Figure 2E. This trace does not look very rhythmic. This might be easier to appreciate if the EMG trace was further smoothed.

Figure 7. The immuno is hard to appreciate and it seems as if the fix might not have been as effective as would like. At the minimum here I'd zoom in on structures as they are very difficult to see.

Reviewer 2:

In this manuscript, the authors studied whether thermo-sensory pathways are functional at birth in a marsupial mammal. They demonstrate in a semi intact preparation, that newborn opossums produce forelimb movements in response to ejection onto the snout of a small quantity of saline at various temperatures, the cold one being much more efficient to evoke motor activity than temperate or not liquids. In an attempt to characterize the receptor responsible for thermal (cold) sensitivity, they investigated but failed to observe the expression of the mammalian principal cold-sensitive receptor TRPM8 at early postnatal days.

The study is generally interesting, since little is known about thermo-sensation development, especially in terms of thermo-sensory behaviors. However, results are often difficult to read, contain many ambiguities and raise several concerns about methodology and analysis. I suggest a thorough rewriting of the result section, in order to simplify the presentation of results (e.g., EMG results are hard to follow: why not give simply the number of failures in each conditions and only compare actual responses, rather than doing a first analysis including all data and saying after that is it not the best way to do it?) and discuss more functional considerations. I believe that it would be of great benefit to focus results and discussion on the thermo-sensory-motor pathway that is evoked in the introduction.

GENERAL COMMENTS

As a general point, numbers of preparations/stimulations/limbs used in the experiments/analyses are not consistent, especially between text and figures, or really require clarification. For example, in EMG experiments, it is written page 14 that 8 specimens were stimulated 10 times with two distinct solutions (4 and 22{degree sign}C), which should correspond to 80 stimulations par temperature; yet in Figure 5-1 A (which corresponds to this part of the results), 117 stimulations are indicated for each temperature. Supposedly, the surplus corresponded to stimulations after surgeries but this was never explained in the text or the corresponding numbers given. Another striking example, page 15, 2nd paragraph, 30 forelimbs were stimulated with neutral temperature... on 8+6=14 animals according to the text pages 14-15. Similarly, numbers of stimulations given for EMG response latency analysis are incorrect or badly explained (page 15, last paragraph): 14 “intact” animals stimulated 10 times with 4{degree sign}C solution gives 140 stimulation max, and only 97% of stimulation evoked a response; the total of stimulation is surely not 213. And it is the same for the two other temperatures used.

Material and Methods indicate that all animals were anesthetized by hypothermia, and I wonder to which extent this protocol could have impacted the results. Several studies on pain, allodynia, etc, report that a cold experience sometimes generates hypersensitivity to following cold exposures that can become noxious. To my understanding, the authors did not want to use noxious stimuli in their study, but rather, wanted to place their work in the context of temperature-related behaviors (as indicated by the reference to Langworthy 1928). Can such sensitization be involved in/responsible for the observation of greater responses to stimuli colder than the medium? Could the authors consider trying other anesthetic protocol(s) to exclude any thermal sensitization and cold-induced pain?

In the same vein, why the various temperatures were always applied in ascending order? Cannot one suppose any order effect?

The categorization into uncoordinated and rhythmic locomotor responses based on behavioral observation is not convincing (according to images of figure 1, and supplemental videos). The difference is not striking. Furthermore, most of the forelimb responses look related more to a startle reaction than to an actual thermo-sensory locomotor behavior. In addition, it is not always clear in the text to which type of movement the authors are referring. We have to wait for page 13 (4th page of results) to understand that the authors are now focusing (in the unique paragraph?) on rhythmic movements. Unfortunately, these are probably the most interesting ones, with comprehensive effects of CNS lesions. Especially, these rhythmic movements were totally abolished after spino-encephalic disconnection (page 13, 2nd paragraph). The results would surely benefit from a clear separation between putative startles and locomotor-like activities.

In contrast, it is hardly conceivable that snout stimulation (whatever the type) still generate limb movements after a complete transection at the obex, whereas the authors report it was the case for most of forelimb movements (top of page 11) and EMG responses (page 14, 2nd paragraph). These latter results clearly indicate that stimulation was not restricted to the snout but probably spread and activated forelimb skin receptors too (corroborated by the very small effect of a 10µL ejection onto the snout, which probably did not diffuse enough to activate skin receptors away from the ejection site; page 13 bottom). In the general context of thermal sensitivity, this would not be a big issue but to my understanding, the authors' aim was to analyze a pathway involving trigeminal afferents specifically. This should be at least discussed. Similarly, the authors should discuss the possible receptor locations. Several works have demonstrated the presence of temperature-sensitive receptors in structures different from the skin (e.g., muscles and mandibular periosteum). Could it be that such receptors may have been involved after skin removal?

MINOR COMMENTS

Page 3, line 10: remove “CPG”. Not used anymore in the manuscript.

Page 3, line18: correct the reference into “Williams-Blangero”.

Page 3, line 22: suppress “small size” (repeated twice).

Page 4, line 6: Not sure the reference to Shankland 2000 is useful.

Page 8, lines 2 and 21: were the immunochemistry and PCR performed on the same animals as those used as semi-intact preparations? If so, please explain how they were chosen. If not, explain why it was not so? Maybe it would have been interesting to perform these analyses on the most responding animals.

Page 9 top and refs: please complete the reference to Majhi et al. in reference list with volume and item numbers (“3:e1310”).

Page 10, line 12: the neutral temperature indicated here (22{degree sign}C) is different from that in figures 3 and 3-1 and table 2 (25{degree sign}C) to which this part of the results refers.

Page 12, last two lines: authors present 25{degree sign}C as the neutral temperature for this series of experiments; however, according to figures it was the case from the beginning (see preceding minor point).

Page 13, line 10: change reference to Fig. 2C by Fig. 2E.

Page 13, line 11-12: please add bibliography reference about newborn opossum behavioral performances.

Page 15, lines 7-8: I don't understand the interest of comparing the responses to hot stimulation before and to neutral stimulation after trigeminal nerve transection. Ok it is given by the post-test statistical analysis but what is the point here?

Page 16, line 8: reference is made to Table 2K, which does not exist.

Page 18, line 4 from bottom: add bracket between “absence” and “i.e., amplitude=0)”.

Page 19, line 2: I guess unity is “ms” rather than “s” for s.e.m.

Page 19, line 2 from bottom: change “suggests” for “to suggest”.

Page 20, end of 1st paragraph: I don't understand the point made that cold should stimulate mainly excitatory circuits in newborn opossums since the authors discussed above that in immature mammals, “inhibitory” circuits also are excitatory before the chloride gradient changes. Or, do the authors mean that newborn opossums already exhibit mature inhibitory systems?

Figure 2 legend: Please specify that bilateral responses are illustrated in C-E.

Table 2J: what is the meaning of the little “d” after “right and left FL”?

Table 2 and Fig.3-1: from abbreviations, please remove supernumerary “skin, facial skin removal”.

Figure 4-1: how the response to a single ejection could be both uncoordinated (99.3% of evoked movements at 4{degree sign}C) and rhythmic (61.8% of movements at 4{degree sign}C)? Same is true for 4{degree sign}C-5N and 4{degree sign}C-obex conditions, with summed ratios exceeding 100%. Here again, it appears that the distinction was not clear, even for the authors.
