# Supplementary material for: Influence of Temperature on Motor Behaviors in Newborn Opossums (Monodelphis domestica): An In Vitro Study
Source: eNeuro. 2019 Jun 4;6(3):ENEURO.0347-18.2019. doi: 10.1523/ENEURO.0347-18.2019 (PMC6553572; doi:10.1523/ENEURO.0347-18.2019)
Supplement: Extended Data Figure 3-1 — FL response rates for behavioral observation experiments. Download Figure 3-1, DOCX file. [file sup_enu-eN-NWR-0347-18-s01.docx]

Figure 3-1. FL response rates for behavioral observation experiments.

|  | Figure | Experimental condition | Number of specimens | Total number of stim | Mean (%) | s.e.m. (%) |
| --- | --- | --- | --- | --- | --- | --- |
| A | 3A | 4°C | 13 | 130 | 100.0 | 0.0 |
|  |  | 21°C | 8 | 80 | 92.5 | 4.1 |
|  |  | 25°C | 13 | 130 | 9.2 | 3.3 |
|  |  | 34°C | 13 | 130 | 8.5 | 3.2 |
|  |  |  |  |  |  |  |
| B | 3B | 4°C | 13 | 130 | 100.0 | 0.0 |
|  |  | 4°C -5N | 5 | 50 | 62.0 | 21.5 |
|  |  | 4°C -obex | 5 | 50 | 30.0 | 18.4 |
|  |  |  |  |  |  |  |
| C | 4A | 4°C | 9 | 90 | 100.0 | 0.0 |
|  |  | 22°C | 9 | 90 | 24.4 | 5.6 |
|  |  | 45°C | 9 | 90 | 37.8 | 11.0 |
|  |  | 4°C | 9 | 90 | 100.0 | 0.0 |
|  |  | 4°C -obex | 9 | 90 | 80.0 | 8.8 |
|  |  |  |  |  |  |  |
| D | 4B | 4°C | 12 | 120 | 97.5 | 1.8 |
|  |  | 22°C | 12 | 120 | 21.7 | 6.9 |
|  |  | 45°C | 12 | 117 | 24.9 | 6.8 |
|  |  | 4°C –skin | 12 | 120 | 89.2 | 6.1 |
|  |  | 22°C –skin | 12 | 120 | 4.2 | 3.4 |
|  |  | 45°C –skin | 12 | 120 | 10.0 | 5.2 |
|  |  | 4°C –obex | 12 | 120 | 23.3 | 10.3 |
|  |  |  |  |  |  |  |
| E | N/A | 4°C (isoflurane) | 4 | 40 | 97.5 | 2.5 |
|  |  | 22°C (isoflurane) | 4 | 40 | 12.5 | 4.8 |
|  |  |  |  |  |  |  |
| F | N/A | 4°C 10 µl | 4 | 40 | 100.0 | 0.0 |
|  |  |  |  |  |  |  |
| G | N/A | 4°C skull | 4 | 40 | 5.0 | 3.5 |
|  |  |  |  |  |  |  |

Abbreviations: stim, stimulations; -5N, trigeminal transection; -obex, complete transection of the spinoencephalic junction, caudal to the obex; -skin, facial skin removal.; N/A, non-applicable; isoflurane, specimens were anesthetized with isoflurane; 10 µl, ejection of 10 µl of 4°C solution; skull, stimulations directed towards the inside of the skull, to the brainstem and 5G.
