# Supplementary material for: Influence of Temperature on Motor Behaviors in Newborn Opossums (Monodelphis domestica): An In Vitro Study
Source: eNeuro. 2019 Jun 4;6(3):ENEURO.0347-18.2019. doi: 10.1523/ENEURO.0347-18.2019 (PMC6553572; doi:10.1523/ENEURO.0347-18.2019)
Supplement: Extended Data Figure 4-1 — FL responses - either all responses (uncoordinated + rhythmic) or rhythmic responses only - induced by temperature in 34 in vitro preparations of newborn opossums in proportion of total stimulations. Download Figure 4-1, DOCX file. [file sup_enu-eN-NWR-0347-18-s02.docx]

Figure 4-1. FL responses – either all responses (uncoordinated + rhythmic) or rhythmic responses only – induced by temperature in 34 *in vitro* preparations of newborn opossums in proportion of total stimulations.

| Bath T° | Stim T° | N (stim) | Movement (uncoordinated +rhythmic) | % | Rhythmic | % |
| --- | --- | --- | --- | --- | --- | --- |
| 22-25 °C | 4 °C | 440 | 437 | 99.3 | 272 | 61.8 |
| 22 °C | 21 °C | 80 | 74 | 92.5 | 20 | 25.0 |
| 22-25 °C | 22-25 °C | 350 | 62 | 17.7 | 6 | 1.7 |
| 25 °C | 34 °C | 130 | 11 | 8.5 | 0 | 0.0 |
| 22 °C | 45 °C | 217 | 69 | 31.8 | 1 | 0.5 |
| 25 °C | 4 °C -5N | 50 | 40 | 80.0 | 18 | 36.0 |
| 22-25 °C | 4 °C- obex | 90 | 72 | 80.0 | 0 | 0.0 |
| 22 °C | 4 °C - skin | 120 | 107 | 89.2 | 19 | 15.8 |
| 22 °C | 22 °C - skin | 120 | 5 | 4.2 | 0 | 0.0 |
| 22 °C | 45 °C - skin | 120 | 12 | 10.0 | 1 | 0.8 |

Abbreviations: Bath T°, bath temperature; Stim T°, stimulation temperature; n, number of stimulations; -5N, trigeminal nerve transection; -obex, complete transection of the spinoencephalic junction, caudal to the obex; -skin, facial skin removal.
