# Supplementary material for: Influence of Temperature on Motor Behaviors in Newborn Opossums (Monodelphis domestica): An In Vitro Study
Source: eNeuro. 2019 Jun 4;6(3):ENEURO.0347-18.2019. doi: 10.1523/ENEURO.0347-18.2019 (PMC6553572; doi:10.1523/ENEURO.0347-18.2019)
Supplement: Extended Data Figure 5-1 — Amplitude of EMG responses to different temperatures. Download Figure 5-1, DOCX file. [file sup_enu-eN-NWR-0347-18-s03.docx]

Figure 5-1. Amplitude of EMG responses to different temperatures.

|  | Figure | Experimental condition | Number of specimens | Number of limbs | Total number of stim | Mean (%) | s.e.m. (%) |
| --- | --- | --- | --- | --- | --- | --- | --- |
| A | 6A | 4°C | 13 | 21 | 201 | 56.6 | 2.0 |
|  |  | 22°C | 13 | 21 | 290 | 5.8 | 0.8 |
|  |  | 45°C | 5 | 9 | 89 | 13.5 | 4.2 |
|  |  | 4°C -5N | 7 | 10 | 100 | 23.7 | 3.0 |
|  |  | 22°C -5N | 12 | 17 | 170 | 0.3 | 0.2 |
|  |  | 45°C -5N | 5 | 7 | 70 | 1.8 | 0.8 |
|  |  | 4°C -obex | 7 | 10 | 88 | 6.3 | 1.5 |
|  |  |  |  |  |  |  |  |
| B | 6B | 4°C | 13 | 21 | 201 | 56.6 | 2.0 |
|  |  | 4°C >0 | 13 | 21 | 194 | 58.7 | 1.9 |
|  |  | 22°C | 13 | 21 | 290 | 5.8 | 0.8 |
|  |  | 22°C >0 | 13 | 21 | 67 | 25.2 | 2.1 |
|  |  | 45°C | 5 | 9 | 89 | 13.5 | 4.2 |
|  |  | 45°C >0 | 5 | 9 | 29 | 41.4 | 11.2 |
|  |  |  |  |  |  |  |  |
| C | N/A | 4°C right FL | 8 | 16 | 69* | 53.4 | 3.5 |
|  |  | 4°C left FL | 8 | 16 | 69* | 61.2 | 3.2 |

Abbreviations: stim, stimulations; -5N, trigeminal transection; -obex, complete transection of the spinoencephalic junction, caudal to the obex; -skin, facial skin removal.; FL, forelimb; N/A, non-applicable; * total number of stimulations are considered for each pairs of limbs.
