# Supplementary material for: Influence of Temperature on Motor Behaviors in Newborn Opossums (Monodelphis domestica): An In Vitro Study
Source: eNeuro. 2019 Jun 4;6(3):ENEURO.0347-18.2019. doi: 10.1523/ENEURO.0347-18.2019 (PMC6553572; doi:10.1523/ENEURO.0347-18.2019)
Supplement: Extended Data Figure 6-1 — Latency of FL responses after temperature stimulations for EMG recordings. Download Figure 6-1, DOCX file. [file sup_enu-eN-NWR-0347-18-s04.docx]

Figure 6-1. Latency of FL responses after temperature stimulations for EMG recordings.

|  | Figure | Experimental condition | Number of specimens | Number of limbs | Total number of stim | Mean (ms) | s.e.m. (ms) |
| --- | --- | --- | --- | --- | --- | --- | --- |
| A | 7 | 4°C | 13 | 21 | 194 | 741 | 27 |
|  |  | 22°C | 13 | 21 | 67 | 1059 | 68 |
|  |  | 45°C | 5 | 9 | 29 | 2303 | 185 |
|  |  |  |  |  |  |  |  |
| B | N/A | 4°C right FL | 8 | 16 | 69* | 842 | 57 |
|  |  | 4°C left FL | 8 | 16 | 69* | 725 | 38 |

Abbreviations: stim, stimulations; FL, forelimb; N/A, non-applicable; * total number of stimulations are considered for each pairs of limbs.
